# Effectiveness of 0.05% cyclosporine A eye drops for dry eye disease in patients with thyroid-associated ophthalmopathy: a prospective, parallel-group cohort study

**DOI:** 10.3389/fmed.2026.1951598

**Published:** 2026-10-07

**Authors:** Yu Zhang, Juan Bao, Cuiyu Wang, Ruiyao Gao, Kai Zhang, Yingxin Chen

**Affiliations:** Department of Ophthalmology, General Hospital of Northern Theater Command, Shenyang, China

**Keywords:** cyclopsporine A, dry eye disease, OSDI, SIT, thyroid-associated ophthalmopathy

## Abstract

**Background:**

Thyroid-associated ophthalmopathy (TAO) is frequently accompanied by dry eye disease (DED), which can lead to ocular surface damage and decreased visual quality. This study aimed to evaluate the effectiveness of 0.05% cyclosporine A (CsA) eye drops in the treatment of TAO-related DED.

**Methods:**

This prospective, parallel-group cohort study included 86 participants diagnosed with TAO-related DED. Participants were divided into either the CsA group (*n* = 43) or the control group (*n* = 43). The CsA group received 0.05% CsA eye drops (twice daily), and control group received polyethylene glycol eye drops (four times daily). Ocular parameters were assessed pre-treatment and at 1, 2, and 3 months post-treatment.

**Results:**

After 3 months of treatment, the CsA group demonstrated significantly better outcomes than the control group in ocular surface disease index (OSDI) scores, fluorescein tear breakup time (FBUT), and Schirmer I test (SIT) values (all *P* < 0.05), with between-group differences emerging as early as month 1. Corneal fluorescein staining (CFS) scores also showed significantly greater improvement in the CsA group (*P* < 0.05). Both groups exhibited comparable improvements in tear meniscus height (TMH) and ocular redness scores over time (*P* > 0.05). The incidence of adverse events was low in both groups, with all events being mild.

**Conclusion:**

0.05% CsA eye drops were associated with improvement in tear film stability, tear secretion, and corneal epithelial integrity in participants with TAO-related DED, and were well tolerated over the 3-month follow-up period.

## 1 Introduction

Thyroid-associated ophthalmopathy (TAO), also known as Graves’ ophthalmopathy, is an autoimmune disorder that primarily affects the retrobulbar tissues (1). As one of the most common orbital disorders in adults, its prevalence reaches 25%–58% among patients with Graves’ disease (1, 2). TAO presents with a wide spectrum of clinical features, progressing from mild symptoms such as eyelid retraction and ocular discomfort to severe complications like corneal exposure, optic nerve compression, and proptosis (3). These manifestations not only significantly impair the patient's appearance and mental health but also pose a substantial risk to vision and quality of life (4). Ocular surface symptoms may occur in the context of diverse immune-mediated and treatment-related inflammatory disorders. Clinical observations from patients with nivolumab-induced uveitis demonstrate that immune dysregulation can produce clinically significant ocular inflammation requiring careful diagnosis and individualized management (5). This broader clinical context is relevant to TAO, in which ocular surface discomfort may coexist with inflammatory orbital and ocular abnormalities. Studies have shown that dry eye disease (DED) is commonly observed in patients with TAO, with approximately 65%–85% of TAO patients also experiencing comorbid DED (6). DED is a multifactorial ocular disorder caused by abnormal tear composition, insufficient tear production, impaired tear circulation, and tear film instability (7). Clinically, the TFOS DEWS III diagnostic and classification system has shifted from the traditional dichotomy of aqueous deficiency and evaporative DED to an etiology-driven framework, which identifies specific drivers including tear film deficiencies, eyelid abnormalities, and ocular surface abnormalities (8). DED is clinically characterized by symptoms such as visual impairment, dryness, foreign body sensation, photophobia, and reflex tearing. If left untreated, the condition may progress to an irreversible chronic inflammatory state, resulting in persistent ocular surface damage and severe, long-term functional impairment, which significantly compromises patients’ quality of life (9, 10). Therefore, exploring safe and effective therapeutic strategies to alleviate symptoms and protect ocular surface structures has become a key focus in current clinical practice.

Currently, the management of TAO-related DED encompasses a multifaceted approach aligned with the TFOS DEWS III management framework (11). Tear supplementation remains the cornerstone of therapy, with viscosity-enhancing agents such as polyethylene glycol, carboxymethylcellulose, and hyaluronic acid addressing aqueous-deficient components, while lipomimetic formulations target evaporative DED associated with meibomian gland dysfunction. However, given that TAO-related DED is fundamentally driven by autoimmune and inflammatory processes, anti-inflammatory therapy—including topical corticosteroids and T-cell immunomodulatory agents such as cyclosporine A (CsA)—is frequently indicated, alongside lifestyle modifications and management of eyelid abnormalities where appropriate (11–13). Beyond conventional artificial tears, advanced ocular lubrication strategies are being investigated to prolong surface hydration and reduce tear-film instability. For example, an injectable copolymer designed to provide *in situ* lubrication has demonstrated potential for relieving DED and improving the ocular surface environment (14). These findings support the importance of sustained lubrication and tear-film stabilization in the management of DED.

CsA, as a classic calcineurin inhibitor, has been widely demonstrated to be effective for moderate to severe DED in its topical eye drop formulation (0.05%) (15, 16). CsA directly targets the immune-inflammatory processes on the ocular surface by inhibiting T-lymphocyte activation and proliferation, thereby reducing the release of key pro-inflammatory mediators such as IL-2 and TNF-α (17). This mechanism aligns with the pathophysiology of TAO-related DED. Moreover, CsA promotes goblet cell proliferation, increases mucin secretion, inhibits apoptosis of corneal and conjunctival epithelial cells, and may improve lacrimal gland secretory function (18). These effects support its status as an ideal candidate drug for treating TAO-related DED. Currently, research on the treatment of TAO-related DED remains relatively limited, particularly regarding the comparative efficacy of 0.05% CsA eye drops versus polyethylene glycol eye drops in improving tear film stability among TAO patients. Polyethylene glycol is a commonly used viscosity-enhancing agent in ophthalmic lubricants; its high molecular weight and hydrophilic properties enable it to increase tear film viscosity and retention time on the ocular surface, thereby providing lubrication and temporary relief of dry eye symptoms (19). To our knowledge, no study has specifically examined the Chinese population or used polyethylene glycol eye drops as a contemporary control. Addressing this research gap, this study aims to systematically compare the clinical outcomes of these two medications in patients with TAO-related DED, thereby providing more effective intervention strategies and theoretical foundations for managing dry eye in this population.

## 2 Materials and methods

### 2.1 Study design

This prospective, parallel-group cohort study included a total of 86 participants diagnosed with TAO-related DED (86 eyes) from the Department of Ophthalmology, General Hospital of Northern Theater Command between June 2025 to November 2025. Participants were divided into the cyclosporine group (CsA group, *n* = 43) and control group (*n* = 43). The study was approved by the Ethics Committee of the General Hospital of Northern Theater Command of the People's Liberation Army [Approval No. Y (2025) 314], and all participants provided written informed consent before participation. In this study, treatment allocation was not at the discretion of the investigators but was determined by clinical practice and patient preference. Therefore, this study does not meet the ICMJE criteria for a clinical trial and is not mandatorily required to be registered as a clinical trial.

### 2.2 Participants

According to the Bartley diagnostic criteria (20), the diagnosis of TAO requires meeting one of the following conditions (1): Presence of eyelid retraction, accompanied by at least one of the following: thyroid dysfunction, exophthalmos, optic neuropathy or extraocular muscle involvement; (2) In the absence of eyelid retraction, the participant must have both thyroid dysfunction and at least one of the following: exophthalmos, optic neuropathy or extraocular muscle involvement, with other diseases causing similar ocular signs being excluded.

Participants meeting the following criteria were included in this study: aged 18 to 70 years; diagnosed with TAO according to Bartley diagnostic criteria and with a Clinical Activity Score (CAS) < 3; simultaneously, participants were newly diagnosed with DED (new-onset, untreated)and met the diagnostic criteria for DED as defined by the Chinese expert consensus on the diagnosis and treatment of dry eye (2024) (21). All included participants had not used any systemic or topical medications for TAO or DED within the 6 months prior to the study and provided written informed consent to participate in at least a 3-month follow-up period. In participants who met the eligibility criteria in both eyes, the eye exhibiting more severe dry eye manifestations was designated as the study eye for statistical analysis. This determination was based on a composite clinical assessment integrating objective parameters at baseline: Schirmer I test (SIT), fluorescein tear breakup time (FBUT), corneal fluorescein staining (CFS), and ocular surface disease index (OSDI). In cases where both eyes demonstrated identical severity, the right eye was consistently chosen for analysis.

Conversely, participants were excluded if they had any of the following conditions: pre-existing DED before the onset of TAO (physician-diagnosed dry eye or persistent symptoms requiring artificial tears before TAO onset, determined by standardized baseline interview and verified by records where available); a history of ocular surgery or trauma; acute ocular surface infections or inflammation; comorbid severe hepatic, renal, or cardiovascular dysfunction; concomitant ocular diseases such as congenital alacrima, glaucoma, or iritis; systemic or immune-mediated diseases (other than thyroid dysfunction) that could cause DED; pregnancy or lactation; allergies to cyclosporine, polyethylene glycol, propylene glycol, or other components of the study medication; history of herpes simplex virus (HSV) keratitis or other contraindications to CsA use; history of contact lens wear during or prior to the study period; or concurrent participation in other clinical trials during the follow-up period.

### 2.3 Study protocol

Prior to treatment, all participants underwent detailed medical history collection and routine ophthalmic examinations (including visual acuity, refraction, intraocular pressure, slit-lamp examination, and anterior segment photography) to exclude contraindications. Subsequently, the same ophthalmologist performed baseline examinations in identical conditions (darkened, draft-free examination room) using consistent equipment (slit lamp, comprehensive ocular surface analyzer, etc.), following a non-invasive-then-invasive sequence.

The CsA group received 0.05% Cyclosporine Eye Drops (II) (specification: 0.4 mL: 0.2 mg; Sinqi Pharmaceutical, Shenyang, China) via topical administration, with a dosage regimen of one drop per dose twice daily (every 12 h). The control group was administered Polyethylene Glycol Eye Drops (40 mg/10 mL; General Saino Pharmaceutical, Shanghai, China) at a dosage of one drop per dose four times daily (22). Treatment was discontinued after three consecutive months of continuous administration for both groups. Adopting a fixed QID regimen for the control group maintained consistency in dosing frequency and minimized the confounding effect of differential instillation frequency on patient adherence and perceived treatment benefit, while avoiding the variability inherent in as-needed use. Participants returned for follow-up visits at 1, 2, and 3 months after treatment initiation. The same examination sequence and equipment were used for all follow-ups, and changes in relevant indicators were recorded and analyzed.

### 2.4 Outcome measures

A set of standardized evaluations was performed to monitor ocular surface conditions and identify any complications, including OSDI, tear meniscus height (TMH), ocular redness score, FBUT, CFS, SIT, and adverse events (AEs). The primary outcome was SIT, with the others as secondary outcomes. Specifically, the symptom severity was quantified using the OSDI questionnaire, which includes 12 items rated on a 0–100 scale, with higher scores reflecting more pronounced symptoms. TMH was obtained using an ocular surface analyzer (MediWorks Precision Instruments Co., Ltd, Shanghai, China), where a value ≤ 0.2 mm indicated possible aqueous-deficient DED. Furthermore, ocular redness grading and scoring were performed by the same ophthalmologist under a slit lamp based on the Validated Bulbar Redness (VBR) grading scale (23). Grading ranges from 0 to 4, with grades 0–2 indicating normalcy and grade 3 or higher suggesting active inflammation, which is an absolute contraindication for surgery. Redness scores range from 0 to 4, with higher scores indicating more severe hyperemia and tissue edema. Fluorescein sodium ophthalmic strips (Tianjin Jingming New Technological Development Co., Ltd, Tianjin, China) were used to assess FBUT and CFS. For CFS results, the cornea was divided into four quadrants and scored 0–3 based on staining intensity, yielding a total score of 0–12, where higher scores indicate greater corneal damage. Additionally, tear secretion testing is performed using Schirmer strips (without anesthetic), with a wetted length ≤ 5 mm indicating DED. Finally, after 3 months of treatment, efficacy was categorized based on comprehensive improvements in OSDI, FBUT, SIT, and CFS. Clinical effectiveness was classified as markedly effective (OSDI score <13, with FBUT, SIT, and CFS at normal levels), effective (OSDI score decreased from baseline and at least two of the three parameters-FBUT, SIT, and CFS-reached normal levels), and ineffective (failure to meet the above criteria). Total effective rate = (markedly effective + effective)/total number of cases × 100%. Normal levels were defined as follows: FBUT grade 0 (normal, with initial breakup time > 10 s and mean breakup time > 14 s); SIT with wetted length between 10 mm and 15 mm; and CFS score of 0. Given the non-randomized design of this study, this efficacy classification was treated as an exploratory endpoint analysis, and the total effective rate was calculated for descriptive purposes only.

### 2.5 Sample size calculation and statistical analysis

The outcome measure selected for sample size estimation was the SIT. With a two-sided significance level of 0.05 and a power of 90%, the estimated mean ± standard deviation (SD) for SIT is 6.85 ± 2.32 in the CsA group and 8.66 ± 2.15 in the control group. Assuming a 1:1 allocation ratio, the calculated minimum sample size required per group was 34. Accounting for an estimated 20% dropout rate, the final total sample size was determined to be at least 86, with 43 participants allocated to each group.

Statistical analysis was performed using SPSS version 27.0. This study analyzed using the complete case analysis set. For continuous variables, those meeting normality criteria were expressed as mean ± SD; otherwise, median (interquartile range [IQR]) was used. Categorical variables were expressed as the number of cases (percentage). For independent samples, t-tests or Mann–Whitney U tests were used for continuous variables, while *χ*^2^ tests or Mann–Whitney U tests were applied for categorical variables. Between-group comparisons used independent samples t-tests (Welch's correction when needed). Within-group changes from baseline were assessed using paired t-tests. The Holm method was applied to adjust for multiple comparisons across outcomes and time points. The between-group difference in the primary outcome (SIT) was interpreted confirmatorily. All other analyses were considered exploratory. For continuous outcomes (OSDI, FBUT, SIT, TMH), repeated-measures analysis of variance (ANOVA) was used to examine group and time effects, with Greenhouse-Geisser correction applied when the sphericity assumption was not met. Simple effects analysis was conducted when interaction effects were significant. Ordinal categorical outcomes (CFS, ocular redness score) were analyzed using generalized estimating equations (GEE). The primary analysis was based on per-protocol set. A two-sided *P* value < 0.05 was considered statistically significant.

## 3 Results

### 3.1 Baseline characteristics

This study initially included 103 participants (103 eyes) who had TAO-related DED between June 2025 and November 2025. During follow-up, 9 participants were lost to follow-up (3 in the control group and 6 in the CsA group). Moreover, 2 participants in the CsA group discontinued the assigned eye drops and were excluded, while 6 participants in the control group were excluded due to use of other topical eye drops during the follow-up period. Consequently, 86 participants (86 eyes) completed the 3-month follow-up, with 43 eyes in each group (control and CsA). The study flow chart was shown in Figure 1. The mean age of the participants in CsA group was 36.88 ± 8.74 years, and that in control group was 35.77 ± 7.13 years. No statistically significant differences were observed between the two groups in baseline demographic or clinical characteristics, such as gender, age, severity of the disease and related dry eye parameters (all *P* > 0.05, Table 1).

**Figure 1:**
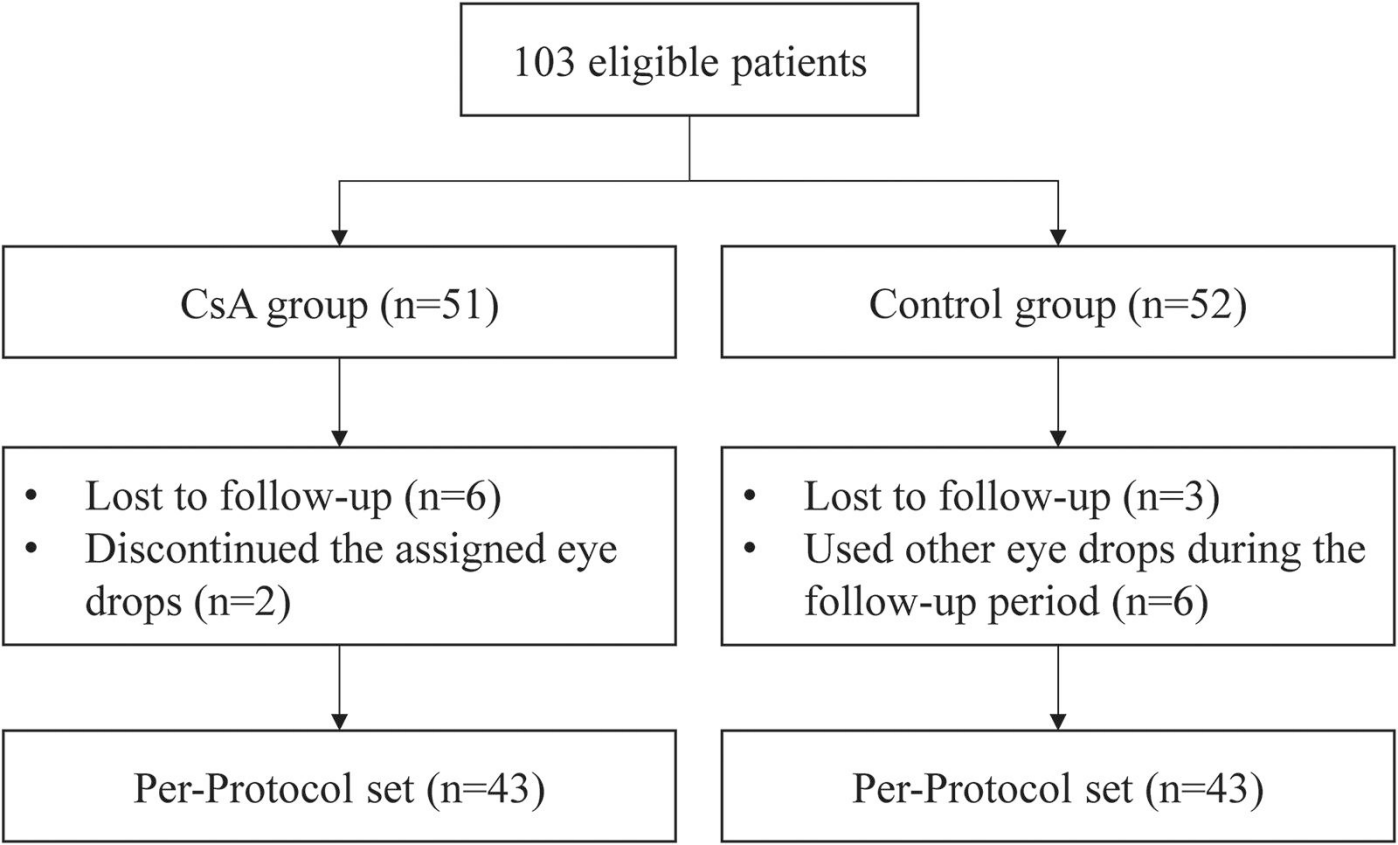
The study flow chart.

**Table 1:** Baseline characteristics of csA and control groups before treatment.

Variable	CsA group (*n* = 43)	Control group (*n* = 43)	*P* value
Gender (*n*, %)			0.486
Male	28 (65.12)	31 (72.09)	
Female	15 (34.88)	12 (27.91)	
Age (years, mean ± SD)	36.88 ± 8.74	35.77 ± 7.13	0.518
Exophthalmos (mm, mean ± SD)	18.34 ± 3.12	18.88 ± 2.15	0.353
MRD-1 (mm, mean ± SD)	5.08 ± 1.03	5.12 ± 0.78	0.806
MRD-2 (mm, mean ± SD)	5.61 ± 1.32	5.58 ± 1.16	0.899
IOP (mmHg, mean ± SD)	19.05 ± 2.88	19.54 ± 3.78	0.500
OSDI (mean ± SD)	26.88 ± 7.32	26.54 ± 6.45	0.820
FBUT (s, mean ± SD)	5.31 ± 1.05	5.23 ± 1.12	0.704
SIT (mm/5 min, mean ± SD)	4.33 ± 1.03	4.32 ± 0.87	0.924
TMH (mm, mean ± SD)	0.15 ± 0.06	0.15 ± 0.07	0.676
CFS (*n*, %)			0.641
2	12 (27.91)	14 (32.56)	
3	31 (72.09)	29 (67.44)	
Ocular redness grade (*n*, %)			0.668
Grade 2	21 (48.84)	23 (53.49)	
Grade 3	22 (51.16)	20 (46.51)	
CAS (*n*, %)			0.293
1	6 (13.95)	3 (6.98)	
2	37 (86.05)	40 (93.02)	
Severity of the disease (*n*, %)			0.309
Mild	3 (6.98)	1 (2.33)	
Moderate-severe	40 (93.02)	42 (97.67)	

SD, standard deviation; OSDI, ocular surface disease index; TMH, tear meniscus height; FBUT, fluorescein tear breakup time; CFS, corneal fluorescein staining; SIT, Schirmer I test; IOP, intraocular pressure; MRD-1, upper lid margin to reflex distance; MRD-2, lower lid margin to reflex distance.

### 3.2 Effectiveness

The repeated-measures ANOVA for the OSDI, FBUT, and SIT revealed a significant interaction effect between group and time (*P* < 0.05). As treatment progressed, OSDI significantly decreased in both groups, while FBUT and SIT significantly increased, indicating substantial improvement in dry eye symptoms (Table 2 and Figures 2A–C). In addition, the CsA group demonstrated more rapid and pronounced improvement in prolonging tear film breakup time and promoting tear secretion compared to the control group (all *P* < 0.05). At the 3-month follow-up, the STI was significantly higher in the CsA group (7.15 ± 1.23) compared to the control group (5.87 ± 0.96, mean difference: 1.28, 95% confidence interval [CI]: 0.81 to 1.75, *P* < 0.001, Supplementary Table 1). Analysis of TMH revealed no statistically significant interaction between group and time (F = 1.903, *P* = 0.139), while the main effect of time was significant (F = 25.844, *P* < 0.001), indicating an overall increase in TMH over time in all participants. Notably, the two groups showed comparable overall improvement in TMH, with no meaningful difference between them (Table 2 and Figure 2D, *P* = 0.341). For CFS score, the interaction effect between group and time, as well as the main effects of group and time, were both statistically significant (*P* < 0.001), indicating that the CsA group was superior to the control group in reducing corneal epithelial damage (Table 2, *P* < 0.05), with time-dependent improvements showing statistically significant differences between groups. For ocular redness score, the interaction between group and time did not reach statistical significance (*P* = 0.636), and the main effect of group showed no evidence of difference (*P* = 0.665). However, the main effect of time was statistically significant (*P* < 0.001), suggesting that both groups displayed comparable trends in reducing conjunctival redness over time, with similar efficacy in alleviating conjunctival hyperemia (Table 2).

**Table 2:** Comparison of dry eye parameters between csA and control groups.

Variable	CsA group (*n* = 43)	Control group (*n* = 43)
OSDI (mean ± SD)		
1-month post-treatment	20.15 ± 4.15 *^a^	22.36 ± 4.32 ^a^
2-month post-treatment	16.41 ± 2.88 **^a^^b^	18.63 ± 3.87 ^a^^b^
3-month post-treatment	11.72 ± 2.29 ***^a^^b^^c^	15.47 ± 3.22 ^a^^b^^c^
FBUT (s, mean ± SD)		
1-month post-treatment	7.32 ± 1.33 ***^a^	6.12 ± 1.31 ^a^
2-month post-treatment	9.68 ± 1.74 ***^a^^b^	7.21 ± 1.54 ^a^^b^
3-month post-treatment	12.39 ± 2.05 ***^a^^b^^c^	8.35 ± 1.87 ^a^^b^^c^
SIT (mm/5 min, mean ± SD)		
1-month post-treatment	5.22 ± 0.86 **^a^	4.66 ± 0.87 ^a^
2-month post-treatment	6.12 ± 1.05 ***^a^^b^	5.13 ± 1.05 ^a^^b^
3-month post-treatment	7.15 ± 1.23 ***^a^^b^^c^	5.87 ± 0.96 ^a^^b^^c^
TMH (mm, mean ± SD)		
1-month post-treatment	0.17 ± 0.08 ^a^	0.18 ± 0.09 ^a^
2-month post-treatment	0.23 ± 0.08 ^a^^b^	0.21 ± 0.09 ^a^^b^
3-month post-treatment	0.28 ± 0.13 ^a^^b^^c^	0.24 ± 0.09 ^a^^b^^c^
CFS (median [IQR])		
1-month post-treatment	2 (1, 3) ^a^	2 (2, 3) ^a^
2-month post-treatment	1 (1, 2) ^a^^b^	2 (1, 2) ^a^^b^
3-month post-treatment	1 (0, 1) *^a^^b^^c^	1 (1, 2) ^a^^b^^c^
Ocular redness score (median [IQR])		
1-month post-treatment	2 (2, 2) ^a^	2 (2, 3) ^a^
2-month post-treatment	2 (1, 2) ^a^^b^	2 (1, 2) ^a^^b^
3-month post-treatment	0 (0, 1) ^a^^b^^c^	0 (0, 1) ^a^^b^^c^

SD, standard deviation; IQR, interquartile range; OSDI, ocular surface disease index; TMH, tear meniscus height; FBUT, fluorescein tear breakup time; CFS, corneal fluorescein staining; SIT, Schirmer I test.

a compared with pre-treatment, *P* < 0.05.

b compared with 1-month post-treatment, *P* < 0.05.

c compared with 2-month post-treatment, *P* < 0.05.

*Compared with control group, *P < 0.05, **P < 0.01, ***P < 0.001.

**Figure 2:**
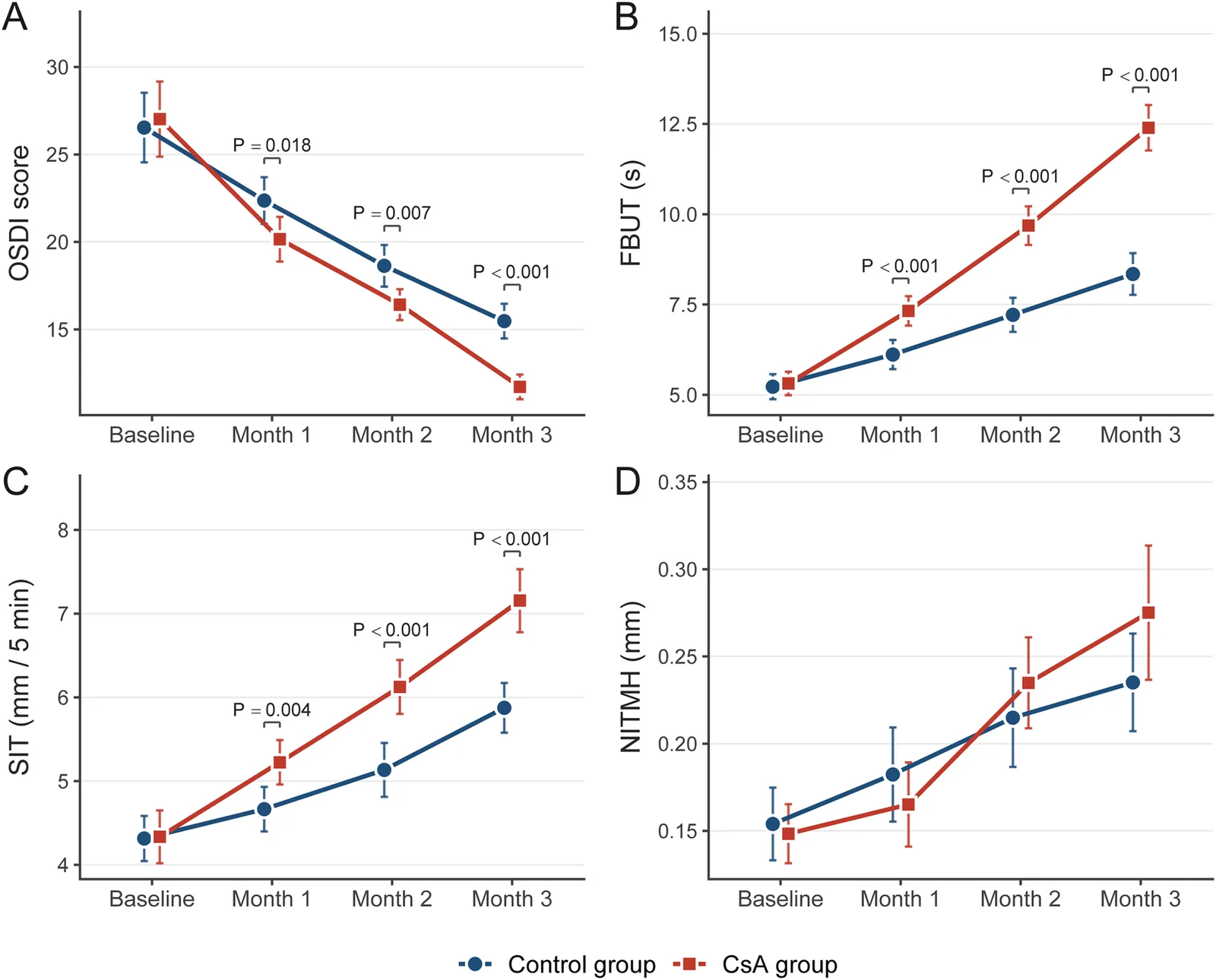
Comparison of dry eye parameters between the two groups. **(A)** Ocular surface disease index (OSDI). **(B)** Fluorescein tear breakup time (FBUT). **(C)** Schirmer I test (SIT). **(D)** Tear meniscus height (TMH). Data are presented as mean ± standard deviation (SD). Error bars represent 95% CI.

According to the efficacy evaluation criteria, 14, 18, and 11 participants in the control group showed ineffective, effective, and markedly effective responses, respectively, yielding a total effective rate of 67.4%. In the CsA group, the corresponding patient numbers were 3, 11, and 29, with a total effective rate of 93.0%. The difference in efficacy grades between the two groups was statistically significant (Z = −4.086, *P* < 0.001), suggesting that the CsA group was associated with better clinical efficacy in this exploratory analysis (Table 3).

**Table 3:** The clinical effectiveness of patients in csA and control groups post-treatment.

Clinical effectiveness, [*n* (%)]	CsA group (*n* = 43)	Control group (*n* = 43)
Markedly effective	29 (67.4)	11 (25.6)
Effective	11 (25.6)	18 (41.8)
Ineffective	3 (7.0)	14 (32.6)
Effective rate	93.0%	67.4%

The overall comparison of efficacy grades between the two groups yielded a Z score of −4.086, with a *P* value of <0.001.

### 3.3 Safety

During the treatment period, AEs were reported in 6 participants (13.95%) in the control group, including blurred vision (*n* = 1), conjunctival hyperemia (*n* = 2), eye irritation (*n* = 2), and eye pruritus (*n* = 1). In the CsA group, 3 participants (6.98%) reported AEs, consisting of conjunctival hyperemia (*n* = 1), eye irritation (*n* = 1), and eye pruritus (*n* = 1). No severe AEs were observed in either group (Table 4).

**Table 4:** Adverse events of patients in csA and control groups post-treatment.

Safety profile, [*n* (%)]	CsA group (*n* = 43)	Control group (*n* = 43)
AEs	3 (6.98)	6 (13.95)
Blurred vision	0 (0.00)	1 (2.32)
Conjunctival hyperemia	1 (2.32)	2 (4.65)
Eye irritation	1 (2.32)	2 (4.65)
Eye pruritus	1 (2.32)	1 (2.32)

## 4 Discussion

This is the first study to use polyethylene glycol eye drops as a control comparator for evaluating the effectiveness of 0.05% CsA eye drops in patients with TAO-related dry eye in a Chinese population. The study indicated that 0.05% CsA eye drops significantly improved clinical symptoms and ocular surface function in patients with TAO-related DED. Compared with the control group, the CsA group was associated with greater improvements in alleviating subjective dry eye symptoms, prolonging tear film breakup time, promoting tear secretion, and repairing corneal epithelial damage, with a faster onset of action. Both groups showed improvement trends in TMH and ocular redness score over treatment duration, but intergroup differences were not statistically significant. Regarding safety, no significant difference in the incidence of AEs was observed between groups. In summary, 0.05% CsA provided an effective and safe treatment option for TAO-related DED.

The existing evidence on CsA for TAO-related DED remains inconclusive. Gürdal et al. reported that 0.05% CsA significantly improved OSDI and TBUT in TAO patients, along with reductions in conjunctival apoptosis and MMP-9 expression (16). However, Altiparmak et al., in a randomized controlled design, found that CsA combined with artificial tears was not superior to artificial tears alone (24). These conflicting findings may reflect differences in study design, sample size, baseline severity, and control selection, underscoring the need for head-to-head comparisons of CsA against contemporary lubricants such as polyethylene glycol eye drops.

The OSDI questionnaire is a widely used standardized assessment tool in ophthalmology for evaluating the severity of dry eye symptoms (25). Our finding showed that OSDI scores decreased significantly in both patient groups over treatment duration, but the CsA group exhibited a greater reduction and achieved the effect earlier. After three months of treatment, the mean OSDI score in the CsA group fell from 26.88 to 11.72 (a 15.16-point reduction), well above the minimal clinically important difference of 7 points (26), a finding that aligns with the clinically meaningful improvements previously reported with CsA in patients with DED unresponsive to artificial tears (27). In TAO patients, ocular surface inflammation often leads to reduced corneal sensitivity and decreased blink rate, creating a vicious cycle of inflammation, hypoesthesia, and worsening dry eye (6). By inhibiting inflammatory cytokines (e.g., IL-1, TNF-α) that sensitize corneal nerve endings, CsA may partially restore corneal sensation, thereby increase blink frequency and improve subjective comfort (28). In the CsA group, FBUT increased from 5.31 s at baseline to 12.39 s at 3 months, compared with an increase from 5.23 s to 8.35 s in the control group. Between-group differences were statistically significant from month 1 onward. This finding was consistent with the observation by Mu et al. in patients with mild DED, where CsA treatment significantly prolonged FBUT after just 4 weeks (29). Moreover, the SIT value in the CsA group increased from baseline (4.33 ± 1.03 mm) to 3 months (7.15 ± 1.23 mm), while the control group only increased from (4.32 ± 0.87 mm) to (5.87 ± 0.96 mm). The difference between groups became significant starting from month 1. These findings suggest that CsA exerted a clear and relatively rapid promoting effect on tear secretion in patients with TAO. Studies have shown that reduced tear secretion in TAO patients is closely associated with CD4+ T cell infiltration in the lacrimal gland (30–32). By inhibiting IL-2 transcription and suppressing T cell activation, CsA alleviates immune inflammation in the lacrimal gland, thereby improving the secretory function of remaining functional acinar cells (33, 34). In this study, between-group difference in SIT values observed as early as one month after treatment provides functional evidence for the efficient clinical translation of CsA's immunomodulatory effects.

TMH reflects the basal volume of tears, which depends on the balance between tear secretion and drainage, evaporation, and the morphology of the eyelid margin. In this study, TMH significantly increased over time, indicating that all patients benefited from artificial tear supplementation and overall improvement in the ocular surface environment. However, the CsA group did not demonstrate superiority over the control group. This outcome may be related to the following factors: First, as a dynamic indicator, TMH is susceptible to factors such as blinking, lighting, and timing of measurement (35), exhibiting considerable measurement variability that may have masked the true effect of CsA. Second, CsA primarily improves tear secretion through anti-inflammatory mechanisms (36), whereas artificial tears directly replenish tear volume. In short-term follow-up, both interventions may yield similar apparent improvements in TMH. Additionally, TAO patients often present with structural alterations such as proptosis and eyelid retraction, which may affect tear distribution and storage on the ocular surface. However, the specific impact of such structural factors on TMH measurements requires further investigation. Furthermore, CFS scores showed that the CsA group was significantly superior to the control group in reducing corneal epithelial damage, with a more pronounced improvement trend over time. Although ocular redness scores decreased over time in both groups, no significant difference was observed between the groups. These findings suggested that CsA provided clear protective effects against T-cell-mediated corneal epithelial immune injury and accelerates the repair of corneal barrier function. In contrast, conjunctival congestion, as a relatively non-specific sign of inflammation, may be influenced by multiple factors such as vascular permeability, impaired venous return, and physical irritation (37, 38); thus, anti-inflammatory treatment with CsA alone may not be sufficient to produce a significant between-group difference. In terms of safety, the incidence of AEs was slightly lower in the CsA group (6.98%) than in the control group (13.95%). All AEs were mild ocular irritation symptoms, which resolved after appropriate symptomatic management, and no systemic AEs were observed. These findings suggested that 0.05% CsA eye drops were well-tolerated and safe in patients with TAO-related DED. Its local irritative symptoms were mild and manageable, without increasing the treatment burden, thereby providing important support for long-term clinical use.

This study has several limitations that should be considered when interpreting the findings. First, the non-randomized, parallel-group observational design precludes causal inference. Treatment allocation was determined by clinical practice and patient preference rather than randomization. Therefore, the two groups may have differed systematically in unmeasured baseline characteristics that could independently influence treatment response, such as disease duration and severity of ocular surface exposure. Additionally, patient expectations and preferences may have influenced perceived symptom improvement, particularly for subjective outcomes such as OSDI. These factors may have biased the results. As a prospective observational cohort study, the primary analysis was performed using the per-protocol set. Although the attrition rate was considered acceptable, participants who discontinued treatment or deviated from the protocol were excluded from the final analysis; therefore, an intention-to-treat analysis was not performed, and the potential for attrition bias cannot be fully excluded. Future studies with larger sample sizes and sensitivity analyses are warranted to confirm our findings. Second, masking was not implemented, which may have introduced performance and detection bias, particularly for subjective assessments such as the OSDI and ocular redness score. Third, the short follow-up period and relatively limited sample size restrict our ability to evaluate long-term efficacy, safety, and recurrence risk. Fourth, the relatively small sample size limits statistical power, particularly for detecting modest effect sizes or infrequent events. We emphasized the exploratory nature of most analyses. Fifth, the CsA group received eye drops twice daily, whereas the control group received polyethylene glycol four times daily. This difference in dosing frequency may have introduced performance bias and could have differentially affected patient adherence or expectations regarding treatment benefit. Sixth, polyethylene glycol is not an inert placebo. The use of an active comparator may have reduced the observed between-group differences, potentially underestimating the true effect size of CsA. Seventh, compliance was assessed via patient-reported diaries and vial counts, without objective measures. Additionally, we did not systematically document adjunct treatments (e.g., warm compresses, lid hygiene) used during follow-up, which may have confounded the results. Eighth, our assessment battery was incomplete. We did not evaluate meibomian gland morphology or function, tear osmolarity, inflammatory biomarkers (MMP-9), or corneal sensitivity, all of which are considered important for comprehensively characterizing TAO-related dry eye. Consequently, our findings primarily reflect tear secretion and corneal epithelial integrity, rather than a complete assessment of ocular surface functional status. Future prospective, randomized controlled trials with larger sample sizes, longer follow-up, blinded outcome assessment, and inclusion of the above-mentioned parameters are warranted to confirm and extend our observations. Additionally, future studies should explore whether patients with more severe or treatment-resistant TAO-related DED require combined local and systemic immunomodulatory approaches. Experiences from other severe immune-mediated disorders, including multicenter evaluations of lymphoplasmapheresis in steroid-refractory neuromyelitis optica spectrum disorder, highlight the importance of real-world evidence and individualized treatment algorithms (39). Objective and reproducible ophthalmic assessment is important for monitoring treatment response in patients with ocular surface and orbital disorders. Recent advances in portable, technology-assisted screening and grading systems, such as PEGSYS, demonstrate the potential value of standardized digital tools for improving the accessibility and consistency of ophthalmic evaluation (40). Future studies could incorporate validated imaging-based or automated assessment methods, such as NIBUT, alongside conventional measures including OSDI, FBUT, SIT, and corneal fluorescein staining.

## 5 Conclusion

In this study population, 0.05% CsA eye drops were associated with significant improvements in clinical symptoms and ocular surface parameters in patients with TAO-related DED, and were well tolerated over the 3-month follow-up period.

## Data Availability

The original contributions presented in the study are included in the article/Supplementary Material, further inquiries can be directed to the corresponding author.
