# Supplementary material for: Effectiveness of 0.05% cyclosporine A eye drops for dry eye disease in patients with thyroid-associated ophthalmopathy: a prospective, parallel-group cohort study
Source: Front Med (Lausanne). 2026 Oct 7;13:1951598. doi: 10.3389/fmed.2026.1951598 (PMC13683219; doi:10.3389/fmed.2026.1951598)
Supplement: Supplementary file 1 [file Table1.docx]

**Supplementary Table 1. Analysis of inter-group comparisons of dry eye parameters at different time points.**

| **Variable** | **CsA group (n=43)** | **Control group (n=43)** | **Mean difference (95% CI)** | ***P* value** | **Adjust-*P* value** |
| --- | --- | --- | --- | --- | --- |
| OSDI (mean ± SD) |  |  |  |  |  |
| pre-treatment | 27.02±6.98 | 26.54±6.45 | 0.48 (-2.40, 3.37) | 0.739 | - |
| 1-month post-treatment | 20.15±4.15 | 22.36±4.32 | -2.21 (-4.03, -0.39) | 0.018 | 0.018 |
| 2-month post-treatment | 16.41±2.88 | 18.63±3.87 | -2.22 (-3.68, -0.76) | 0.003 | 0.007 |
| 3-month post-treatment | 11.72±2.29 | 15.47±3.22 | -3.76 (-4.95, -2.56) | <0.001 | <0.001 |
| FBUT (s, mean ± SD) |  |  |  |  |  |
| pre-treatment | 5.31±1.05 | 5.23±1.12 | 0.09 (-0.38, 0.56) | 0.704 | - |
| 1-month post-treatment | 7.32±1.33 | 6.12±1.31 | 1.21 (0.64, 1.78) | <0.001 | <0.001 |
| 2-month post-treatment | 9.68±1.74 | 7.21±1.54 | 2.47 (1.77, 3.17) | <0.001 | <0.001 |
| 3-month post-treatment | 12.39±2.05 | 8.35±1.87 | 4.05 (3.21, 4.89) | <0.001 | <0.001 |
| SIT (mm/5 min, mean ± SD) |  |  |  |  |  |
| pre-treatment | 4.33±1.03 | 4.32±0.87 | 0.02 (-0.39, 0.43) | 0.924 | - |
| 1-month post-treatment | 5.22±0.86 | 4.66±0.87 | 0.56 (0.19, 0.93) | 0.004 | 0.004 |
| 2-month post-treatment | 6.12±1.05 | 5.13±1.05 | 0.99 (0.54, 1.44) | <0.001 | <0.001 |
| 3-month post-treatment | 7.15±1.23 | 5.87±0.96 | 1.28 (0.81, 1.75) | <0.001 | <0.001 |
| NITMH (mm, mean ± SD) |  |  |  |  |  |
| pre-treatment | 0.15±0.06 | 0.15±0.07 | -0.01 (-0.03, 0.02) | 0.676 | - |
| 1-month post-treatment | 0.17±0.08 | 0.18±0.09 | -0.02 (-0.05, 0.02) | 0.340 | 0.594 |
| 2-month post-treatment | 0.23±0.08 | 0.21±0.09 | 0.02 (-0.02, 0.06) | 0.297 | 0.594 |
| 3-month post-treatment | 0.28±0.12 | 0.24±0.09 | 0.04 (-0.01, 0.09) | 0.093 | 0.281 |

Abbreviations: SD, standard deviation; CI, confidence interval; OSDI, ocular surface disease index; NITMH, non-invasive tear meniscus height; FBUT, fluorescein tear breakup time; SIT, Schirmer I test.
